# Right ventricular myocardial biopsy with a guiding catheter for conduction system pacing during pacemaker implantation revealed transthyretin cardiac amyloidosis

**DOI:** 10.1016/j.hrcr.2024.06.008

**Published:** 2024-06-19

**Authors:** Kei Morishita, Katsuhito Fujiu, Kenichiro Yamagata, Eisuke Amiya, Norihiko Takeda

**Affiliations:** Department of Cardiology, The University of Tokyo Hospital, Tokyo, Japan

**Keywords:** Transthyretin cardiac amyloidosis, Myocardial biopsy, Preshaped guiding catheter, Pacemaker implantation, Tafamidis, Left ventricular hypertrophy, Heart failure with preserved ejection fraction

**Key Teaching Points**

• Transthyretin amyloid cardiomyopathy (ATTR-CM) was diagnosed based on simultaneous biopsies of the right ventricular myocardium and subcutaneous fat during pacemaker implantation, leading to the initiation of tafamidis treatment.
• Myocardial biopsy of the right ventricular septum can be performed safely using a guiding catheter for conduction system pacing.
• Simultaneous biopsies during pacemaker implantation may improve the diagnostic accuracy and therapeutic outcomes of ATTR-CM without additional invasiveness.

## Introduction

Transthyretin amyloid cardiomyopathy (ATTR-CM) is a progressive disease that causes heart failure and arrhythmias, such as sinus node dysfunction, atrial fibrillation, and atrioventricular (AV) block. Disease suspicion usually arises during transthoracic echocardiography, which reveals significant left ventricular hypertrophy (LVH), followed by radionuclide bone scintigraphy with technetium-labeled bisphosphonates.

Effective drug therapies for ATTR-CM have emerged recently; however, their efficacy is limited to early-stage patients.^1^ Therefore, early diagnosis is important but often challenging, especially when bone scintigraphy shows grade 0 (negative) or grade I results, as these cases require myocardial biopsy for a definitive diagnosis. Biopsy of subcutaneous fat as an alternative is less invasive but has a low sensitivity (≈15%).^2^ In contrast, myocardial biopsy has a nearly definitive diagnostic power at the cost of a greater risk of severe complications.^3^ Hence, it is necessary to develop a safe and effective method for myocardial biopsy.

To address this unmet need, we report a case of ATTR-CM confirmed by a novel right ventricular biopsy technique during pacemaker implantation (PMI) using a guiding catheter (GC) for conduction system pacing (CSP).

## Case report

**Figure 1:**
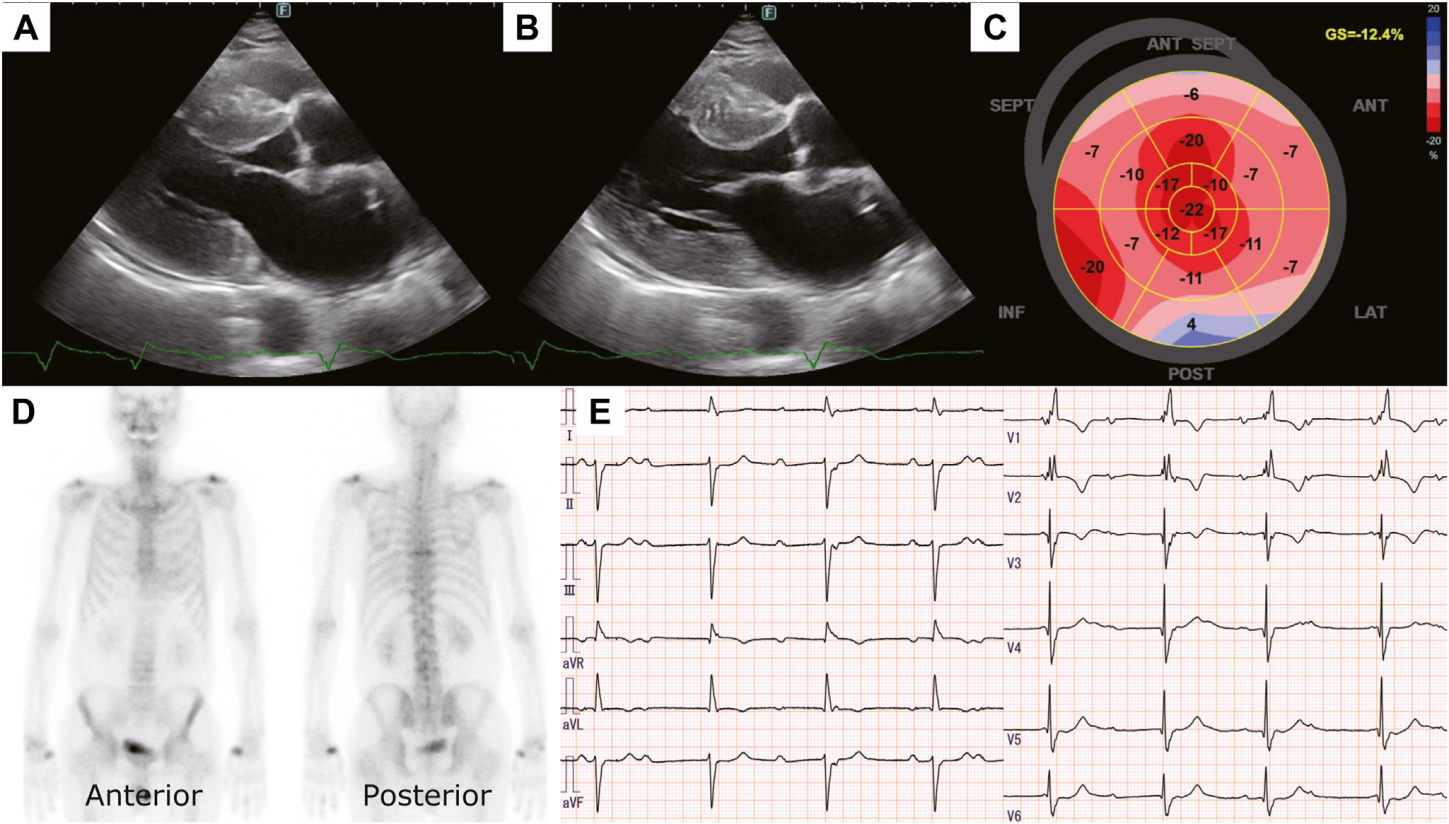
Screening imaging studies. **A, B:** Left ventricular hypertrophy (interventricular septum / posterior wall = 17 / 16 mm) with a normal left ventricular ejection fraction (= 57%) on transthoracic echocardiogram. **C:** Apical sparing pattern of the left ventricular global longitudinal strain, a typical finding in cardiac amyloidosis. **D:**^99m^Tc-hydroxymethylene diphosphonate scintigraphy shows no tracer accumulation to the heart (grade 0). **E:** Complete atrioventricular block with complete right bundle branch block and left axis deviation on 12-lead electrocardiogram.

An 85-year-old man presented to our department for a preoperative cardiac assessment before a prostate biopsy. Transthoracic echocardiography revealed severe LVH with preserved left ventricular ejection fraction (Figure 1A and 1B). Left ventricular global longitudinal strain showed an apical sparing pattern (Figure 1C). Electrocardiography exhibited complete right bundle branch block and left axis deviation with no further abnormalities, and ^99m^Tc-hydroxymethylene diphosphonate scintigraphy depicted the absence of cardiac accumulation (grade 0; Figure 1D), leading to a negative diagnosis of ATTR-CM. No further examinations, including biopsy, were performed, as apparent symptoms of heart failure were not observed.

One year later, the patient was referred to our cardiology department owing to shortness of breath with complete atrioventricular block (CAVB) (Figure 1E) and required PMI. Although the previous bone scintigraphy was negative, the occurrence of CAVB prompted the reconsideration of further evaluation for ATTR-CM, including biopsy.

To minimize invasiveness, we performed biopsies of the right ventricular myocardium using a GC for CSP and subcutaneous fat of the pacemaker pocket during PMI. First, a pocket was created in the left subclavicular chest wall, and a 9 × 5 × 4 mm sample of subcutaneous fat was obtained from inside the pocket using a scalpel. Second, a guide wire was inserted into the left subclavian vein. Third, a preshaped GC (Selectra 3D 40 mm curve; Biotronik, Berlin, Germany), which is usually used for implanting a pacemaker lead for CSP, was threaded into the left subclavian vein over the guide wire and advanced to the right ventricle. Fourth, after removing the guidewire, a bioptome (5F Disposable Biopsy Forceps; Technowood, Tokyo, Japan) was inserted into the GC, which was used for myocardial biopsy. Finally, the bioptome was removed from the GC. The time required for fat biopsy was less than 1 minute and that required for myocardial biopsy was less than 2 minutes. After the biopsies, left bundle branch pacing was initially attempted but failed to penetrate the thick septum; therefore, right ventricular septum (RVS) pacing was finally chosen. The patient was discharged on postoperative day 9 without any complications.

**Figure 2:**
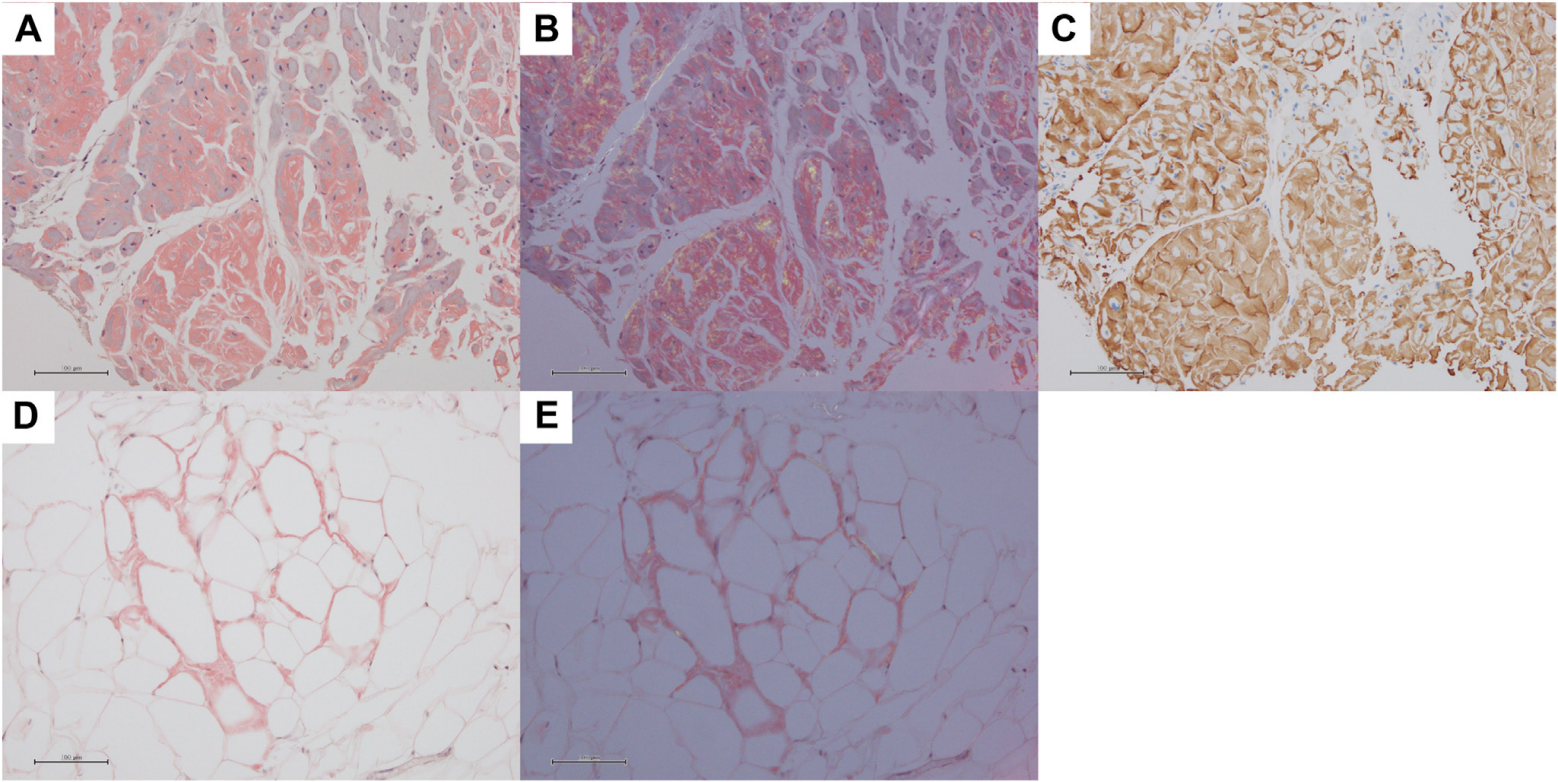
Biopsy samples of right ventricular myocardium and subcutaneous fat at the time of pacemaker implantation. **A:** Direct fast scarlet (DFS) staining of the right ventricular myocardium showed orange amyloid deposits. **B:** DFS staining of the right ventricular myocardium under polarized light showed apple-green birefringence. **C:** Immunohistochemical staining of the right ventricular myocardium showed positive results for transthyretin. **D:** DFS staining of the subcutaneous fat showed orange amyloid deposits. **E:** DFS staining of the subcutaneous fat under polarized light showed the apple green birefringence.

ATTR-CM was confirmed in the myocardial sample following direct fast scarlet staining and apple-green birefringence examination under cross-polarized light microscopy and immunohistochemical staining (Figure 2A–2C). Amyloid deposits were also observed in fat samples using direct fast scarlet staining and apple-green birefringence examination (Figure 2D and 2E). Genetic sequencing of the *TTR* revealed no mutations, resulting in a definitive diagnosis of wild-type ATTR-CM. Following this confirmation, tafamidis was prescribed, and the patient experienced no further episodes of heart failure.

## Discussion

We present a case of ATTR-CM diagnosed using a novel biopsy technique during PMI without increasing invasiveness. The accurate diagnosis enabled the initiation of tafamidis.

In a systematic review by Magdi and colleagues,^4^ up to 11% of patients with heart failure with preserved ejection fraction were diagnosed with ATTR-CM. However, the diagnostic process is complicated, and delayed diagnosis or misdiagnosis often occurs.^5^ In this case report, we suspected ATTR-CM in the early phase; however, the ATTR-CM diagnosis was delayed based on the negative ^99m^Tc-hydroxymethylene diphosphonate scintigraphy results. Myocardial biopsy was initially considered^6^; however, its high invasiveness was a concern owing to the patient’s advanced age and absence of heart failure signs.

Abdominal fat aspiration can be used to diagnose cardiac amyloidosis. Unfortunately, the sensitivity of this diagnostic technique is low, especially in cases of wild-type ATTR-CM (15%).^2^ Fat biopsy from a pacemaker pocket during device implantation has been reported.^7^^,^^8^ Nonetheless, the accuracy of diagnosis remains uncertain; even among patients with clinically suspected ATTR-CM, only 20% tested positive for Congo red staining.^9^

In our case, the patient was strongly suspected of having ATTR-CM owing to the emergence of CAVB, in addition to known LVH; therefore, we performed a myocardial biopsy along with a fat biopsy from the pacemaker pocket during PMI to increase diagnostic sensitivity. Myocardial biopsy carries a risk of serious complications, such as cardiac perforation, which is rare but can be fatal.^3^ Sampling should therefore be performed from the RVS rather than the right ventricular free wall to mitigate the risk of cardiac perforation. Imaging techniques, such as fluoroscopy or 2-dimensional echocardiography, are used to confirm the orientation of the bioptome, but the accuracy of both tests has been questioned.^3^

**Figure 3:**
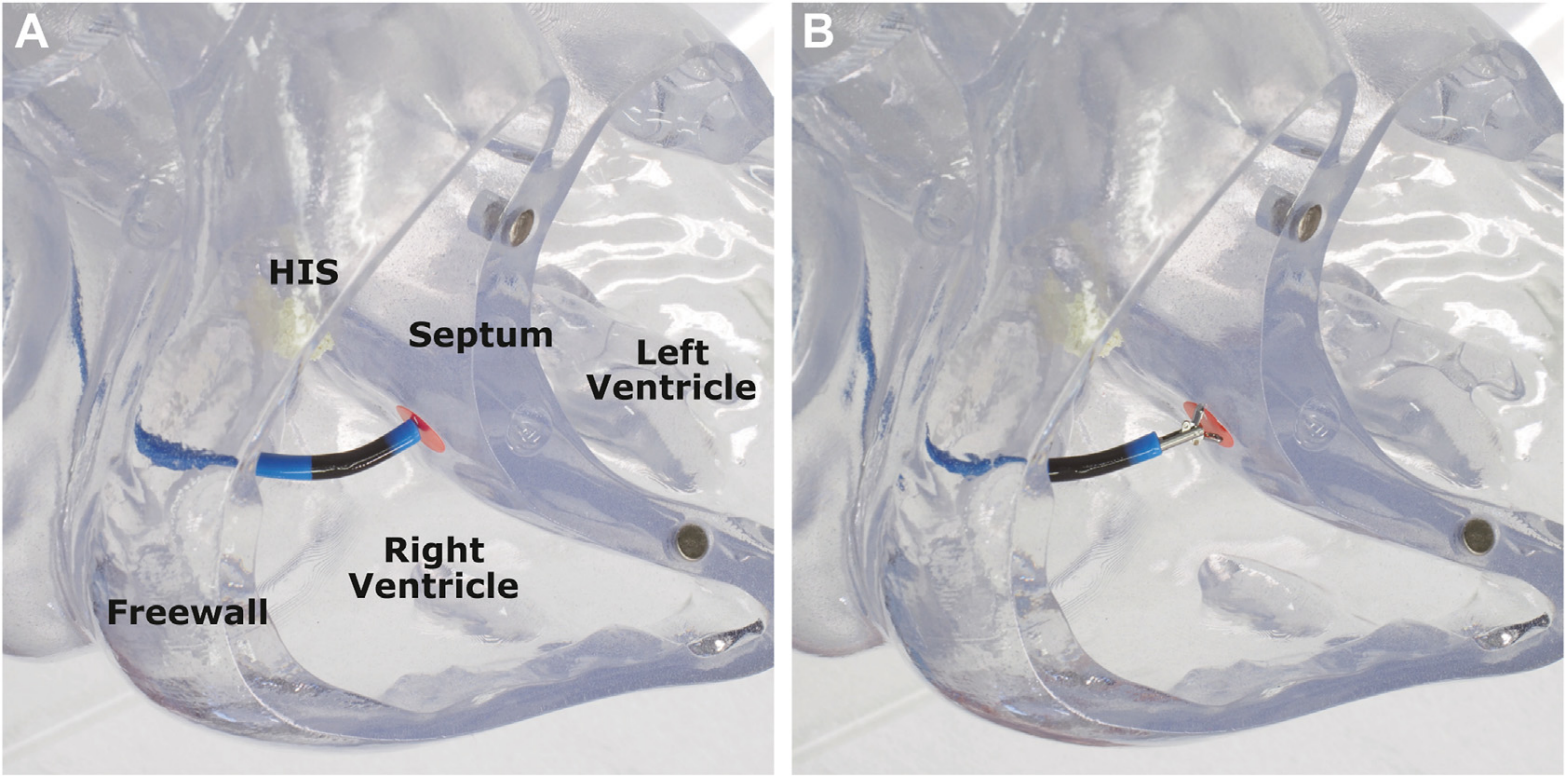
Demonstration of the guiding catheter in a heart model, without and with the bioptome. **A:** The original shape of the guiding catheter (Selectra 3D, 40 mm; Biotronik, Berlin, Germany) without the bioptome shows a 3-dimensional curve directed to the septum. The red mark indicates the right ventricular septum. **B:** Guiding catheter showing that the 3-dimensional curve is still preserved even when the bioptome exits the tip of the catheter. The red mark indicates the right ventricular septum.

We considered using a GC for CSP to address the challenge of confirming the orientation of the bioptome. In a retrospective study for pacing lead implantation, using a GC for CSP achieved 100% accuracy in implanting the lead at the RVS, compared to 44% at the free wall without the use of a catheter.^10^ This GC, originally designed to deliver the pacing lead to the RVS, could also serve the same purpose when delivering the bioptome during myocardial biopsy (Figure 3A and 3B), emphasizing its versatility. Compared to the conventional biopsy approach, the current method may result in a permanent AV block, as it has a higher chance of taking the specimen at the His region owing to the shape of the GC. Therefore, careful biopsy location must be considered to avoid performing the biopsy too proximal in the right ventricle, especially in cases where AV conduction should be preserved.

In the present case, both myocardial and fat biopsies were performed during PMI without significantly interfering with the procedure. Histological results were positive for both in the current study. Stepwise biopsy of the myocardial biopsy after confirming negative fat biopsy could be considered. However, as intraoperative rapid diagnosis is difficult for ATTR-CM, we performed 2 biopsies in succession.

We believe our method may improve the diagnostic accuracy of ATTR-CM without additional invasiveness. Studies are required to confirm the concordance of myocardial and fat biopsies and safety of the simultaneous catheter-guided myocardial biopsy and PMI. Using the GCs for CSP in routine cardiac biopsy practice from superior access may also increase procedure safety. However, this hypothesis needs to be confirmed in future studies.

## Conclusion

ATTR-CM was diagnosed through a simultaneous biopsy of the right ventricular myocardium using a GC for CSP and PMI. This method may improve ATTR-CM diagnostic sensitivity without increasing the risk of complications.

## Disclosures

The authors declare no conflicting interests.
